# Interstitial Lung Disease in Rheumatoid Arthritis in the Era of Biologics

**DOI:** 10.1155/2011/931342

**Published:** 2011-12-20

**Authors:** A. Picchianti Diamanti, V. Germano, E. Bizzi, B. Laganà, A. Migliore

**Affiliations:** ^1^Chair and Division of Allergy, Clinical Immunology, and Rheumatology, S. Andrea University Hospital, School of Medicine and Psychology, Sapienza University of Rome, 00189 Rome, Italy; ^2^Unit of Rheumatology, “S. Peter Hospital FBF” and Research Center “S. Pietro AfaR”, 00100 Rome, Italy

## Abstract

Interstitial lung disease (ILD) represents a severe manifestation in connective tissue diseases (CTD), with an overall incidence of 15%, and it is still a challenge for clinicians evaluation and management. ILD is the most common manifestation of lung involvement in Rheumatoid Arthritis (RA), observed in up to 80% of biopsies, 50% of chest Computed Tomography (CT) and only 5% of chest radiographs. Histopatological patterns of ILD in RA may present with different patterns, such as: usual interstitial pneumonia, non specific interstitial pneumonia, desquamative interstitial pneumonia, organizing pneumonia, and eosinophilic infiltration. The incidence of ILD in RA patients is not only related to the disease itself, many drugs may be in fact associated with the development of pulmonary damage. Some reports suggest a causative role for TNF**α** inhibitors in RA-ILD development/worsening, anyway, no definitive statement can be drawn thus data are incomplete and affected by several variables. A tight control (pulmonary function tests and/or HRCT) is mandatory in patients with preexisting ILD, but it should be also performed in those presenting risk factors for ILD and mild respiratory symptoms. Biologic therapy should be interrupted, and, after excluding triggering infections, corticosteroids should be administered.

## 1. Introduction

Connective tissue diseases (CTD) represent a heterogeneous group of immunologicaly mediated disorders that may affect a wide variety of organs. Respiratory system is frequently involved in some CTD such as rheumatoid arthritis (RA), systemic sclerosis, dermatomyositis, Sjögren syndrome, and undifferentiated CTD [1]. All respiratory components may be interested for the disease: pleura, parenchyma, airways, and vessels. Interstitial lung disease (ILD) is an established clinical corollary with an overall incidence of 15% in CTD; it represents a severe manifestation that is still a challenge for clinicians evaluation and management [2]. It has been postulated that an initial trigger (virus, trauma, etc.) activates resident cells which produce proinflammatory mediators; subsequently chemotactic cytokines guide the infiltration of inflammatory cells which produce profibrotic mediators such as transforming growth factor B (TGF-*β*), platelet-derived growth factor, and IL-4. The matrix component accumulates in the extracellular compartment and disrupts the physiologic structure causing severe functional impairment [3, 4].

## 2. Rheumatoid Arthritis and ILD

Rheumatoid Arthritis (RA) is an inflammatory autoimmune disease that affects approximately 1% of the population potentially leading to functional disability, with about 30% of patients unable to work after 3 years of disease [5]. Nearly 50% of RA patients present extra-articular manifestations involving skin, eye, heart, and lungs [6]. Although cardiovascular disease is responsible for the majority of RA-related deaths, pulmonary complications are frequent and cause 10–20% of overall mortality [7]. ILD is the most common manifestation of lung involvement in RA observed in up to 80% of biopsies, 50% of chest CT, and only 5% of chest radiographs [8].

Mean age at lung disease onset is the fifth or sixth decade; some studies reported an increased prevalence in males and longstanding RA [9, 10]. Furthermore, smokers and patients with rheumatoid nodules, high titre of rheumatoid factor, and antinuclear antibodies are described to be at higher risk of developing ILD, whereas disease severity seems to be an irrelevant factor [11]. Human leukocyte antigen B40 and *α*1 antitrypsin were already described associated with ILD development [12]. The most frequent respiratory symptoms are progressive dyspnea and dry cough; the majority of patients present fine bibasilar crackles, and clubbing is less common than in idiopathic pulmonary fibrosis (IPF) [11]. Histopathological patterns of ILD in RA may present with various manifestations, sometime associated: usual interstitial pneumonia (UIP), nonspecific interstitial pneumonia (NSIP), desquamative interstitial pneumonia (DIP), organizing pneumonia, and eosinophilic infiltration [13]. Data on the different prevalence of reported patterns are contrasting. Lee et al. identified the UIP as the most common histopathological pattern (56%) in contrast with Tansey et al. who described the NSIP as the most prevalent [14–18] Table 1. Imaging data have demonstrated that HRCT is more sensitive than radiography and correlates with histopathological patterns; thus, it may allow for an early diagnosis and should be performed in RA patients manifesting either risk factors for ILD or minor abnormalities on chest radiographs [17]. Recently, Zou et al. reported the most common findings observed with HRCT in 110 RA patients: ground glass (39.9%), honeycombing (4.5%), reticular patterns, and consolidation (1.8%); patients with reticular patterns and honeycombing were more likely to show the respiratory symptoms. Other described abnormalities are architectural distortion, pulmonary nodules, emphysema, bronchiectasis and traction bronchiectasis, subpleural nodules, pleural thickening, and lymph node enlargements [17, 19]. Among routinely exams, pulmonary function tests (PFT) with diffusing capacity for carbon monoxide (DLCO) are the most sensitive; a restrictive defect is typical, but mixed findings may be also present [20].

Despite bronchoalveolar lavage (BAL) may reveal the presence of neutrophil alveolitis and neutrophil percentage and has been reported to correlate with DLCO reduction, it is not usually adopted in ILD-associated RA patients and it is generally used to exclude complicating infections and malignancy [21]. To adopt the correct ILD treatment in RA patients is still a challenge. Ideally therapy should be tailored to the histopathological pattern that remains the best prognostic factor (i.e., the presence of active inflammatory lesions (cellular interstitial pneumonia and lymphoid hyperplasia) that may be reverted by an early treatment).

## 3. ILD and Biological Therapy

The incidence of ILD in RA patients is not only related to the disease itself. Many drugs may be associated with the development of pulmonary damage: nonsteroidal anti-inflammatory drugs, intravenous immunoglobulin, and synthetic DMARDs such us methotrexate, leflunomide, and cyclophosphamide [22]. The introduction of biologic DMARDs has dramatically improved the course of RA by reducing symptoms, arresting structural damage, and thus leading to a significant amelioration of quality of life in these patients [23]. Anyway, these agents are not free from side effects among whom are infections, malignancies, and demyelinating disorders [24, 25]. New onset or ILD worsening has already been reported as a possible consequence of the three TNF*α* inhibitors which are the most widely used class of biologics [26–28] (Table 2): infliximab (a chimeric IgG1k monoclonal antibody consisting of human constant and murine variable regions), Etanercept (a dimeric fusion protein consisting of the extracellular ligand-binding portion of human 75 kDA TNF receptor linked to the Fc portion of human IgG1), and Adalimumab (a recombinant human IgG1 monoclonal antibody specific for human TNF*α*) [29].

In 2002, Peno-Green et al. described the first case of ILD in an RA patient after anti-TNF*α* therapy (Etanercept) [30]. By now, a total of 144 RA patients with new onset or ILD worsening after anti-TNF*α* (55 infliximab, 95 etanercept, and 4 adalimumab) therapy have been reported [31]. In 2010, Dixon et al. showed higher risk of death with a higher prevalence of ILD in the anti-TNF*α* group with respect to synthetic DMARDs (2.8% versus 1.9%  *P* = 0.006) [26].

Alvarez et al. analyzed clinical characteristics, outcomes, and patterns of association of all reported cases of 122 patients (108 adult RA patients) developing ILD after biologic therapy (Etanercept 58, Infliximab 56, adalimumab 3, and Rituximab 5). Patients generally manifested dyspnea, fever and cough, malaise, pleuritic pain, and haemoptysis; the disease onset generally appeared after at least 6 months of therapy. Diagnosis performed by HRCT (36 detailed cases) revealed ground-glass opacities, reticular nodular, nodular pattern, and honeycomb changes; BAL showed lymphocytes and macrophages. Histopathologic findings were UIP, NSIP, organizing pneumonia, and lymphoid interstitial pneumonia. Treatment of ILD included biologic agents withdrawal and the use of corticosteroids, CYC, and azathioprine. The mortality rate was increased by age >65 years, later onset of ILD, and the association of methotrexate and anti-TNF*α* agents [32]. In contrast with these observations, two prospective observational studies have examined the influence of anti-TNF*α* therapy upon ILD. In Japan, 7091 RA patients treated with etanercept and 5000 patients treated with infliximab and methotrexate were monitored for 6 months. Only 0.5% and 0.6% patients were reported to have ILD [27, 28] (Table 2). In addition, other authors reported good results by the use of these agents in patients with RA-ILD. Bargagli et al. showed an improvement in lung function evaluated by PFT DLCO and a stabilization of HRCT abnormalities after infliximab and suggests that it may be considered as a therapeutic option for the treatment of ILD-RA [33]. Similar results are reported by Vassallo et al. [34]. Moreover, Antoniou et al., in 2007 evaluating the potential effectiveness of Infliximab treatment for pulmonary fibrosis in three RA and one SSc patients described that pulmonary fibrosis remained stable during treatment in terms of symptoms, PFTs, and HRCT [35]. These conflicting results may be explained by the complex and dualistic role of TNF*α* and reflect the contradictory data derived by studies *in vitro *and in murine models.

TNF*α* is produced by macrophages, CD4+ and CD8+ T cells, B cells, neutrophils, endothelial cells, and fibroblast. Several studies indicate a profibrotic role for this molecule. Transgenic mice overexpressing murine TNF*α* in the lung develop a chronic lymphocytic alveolitis which severity correlates with the expression of TNF*α* mRNA. Moreover, TNF*α* may upregulate TGFb1 expression in the lungs via the activation of regulated kinase pathway in fibroblasts [36, 37]. On the other hand, mice KO for TNF*α* develop a bleomycin-induced pulmonary fibrosis that may be reverted by the administration of TNF*α* [38]; TNF-*α* is also able to block the synthesis of collagen production and inhibits *α*2 collagen gene transcription in human dermal fibroblasts [39]. Few reports are currently available for the new biologic drugs: rituximab (an engineered chimeric monoclonal antibody directed against the anti-CD20 antigen found on the surface of normal and malignant B cells) and tocilizumab (humanized anti-IL-6 receptor monoclonal antibody). Sixteen cases of rituximab-induced interstitial lung disease have been reported in haematological disorders whereas only two cases of organizing pneumonia have been described in RA patients [40, 41]; a clinical trial on the benefit of rituximab in RA lung disease is ongoing [42]. Kawashiri et al. recently reported a fatal case of acute exacerbation of ILD in a RA patient 10 months after starting tocilizumab [43] No data are currently reported about ILD onset or exacerbation for abatacept (a dimeric fusion protein composed of CTLA4 extracellular domain and human FcIgG1, developed to block the interactions of CD28-CD80/CD86 thus impairs T-cell activation) in RA patients [31]. 

## 4. Conclusions

In conclusions, even if some reports seem to suggest a causative role for TNF*α* inhibitors in RA-ILD development/worsening, no definitive statement can be drawn. In addition these data are affected by several variables (ethnicity, comorbidity, association with synthetic DMARDs, type of study, etc.). A tight control (pulmonary function tests and/or HRCT) is mandatory in patients with preexisting ILD, but it should be also performed in those presenting risk factors for ILD and mild respiratory symptoms. Biologic therapy should be interrupted, and, after excluding triggering infections, corticosteroids should be administered. Larger controlled studies specifically designed to assess the safety profile of anti-TNF-*α* and new biologics in ILD-RA patients at different steps of ILD progression are needed.

## Figures and Tables

**Table 1:** Histopathological patterns of RA-associated ILD.

References	No. of patients	Percentage of different histopathological patterns
Lee et al., 2005 [14]	18	UIP 10 (56%) NSIP 6 (33%) OP (11%) FB 1 (6%) Other 1 (6%)

Tansey et al., 2004 [15]	17	NSIP 8 (47%) FB 6 (35%) UIP 2 (12%) Other 1 (6%)

Bongartz et al., 2010 [16]	46 (14 of whom based on biopsy and/or CT)	UIP 9 (20%) NSIP 1 (2%) OP 3 (6,5%) Other 15 (32.6%)

Tanaka et al., 2004 [17]	63 (17 of whom based on biopsy)	NSIP 10 (59%) UIP 2 (12%) DIP 1 (6%) Other 3 (18%)

Akira et al., 1999 [18]	29 (19 of whom based on biopsy)	UIP 14 (74%) OP 3 (16%) NSIP 1 (5%) Other 1 (5%)

UIP: usual interstitial pneumonia; NSIP: nonspecific interstitial pneumonia; OP: organizing pneumonia; FB: follicular bronchiolitis; DIP: desquamative interstitial pneumonia.

**Table 2:** Number and percentage of RA patients presenting ILD under anti-TNF*α* treatment.

Total no. of RA patients	Patients developing ILD				References
	anti-TNF*α* N/%	Infliximab N/%	Etanercept N/%	Adalimumab N/%	
10.649	299 (2.8%)	UN	UN	UN	Dixon et al., 2010 [26]

13.894	77(0.6%)	UN	77 (0.6%)	UN	Koike et al., 2011 [27]

5.000	25(0.5%)	UN	25 (0.5%)	UN	Takeuchi et al., 2008 [28]

ILD: interstitial lung disease; UN: unavailable.
